# Patients’ Attitudes and Sources of Information on Coronavirus Disease 2019 in Rural Michigan

**DOI:** 10.7759/cureus.14036

**Published:** 2021-03-22

**Authors:** Vaishali Kapila, Ibrahim Baida, Adriana Calderon, Thomas Stuut, Chin-I Cheng, Wendy S Biggs

**Affiliations:** 1 Family Medicine, Central Michigan University College of Medicine, Mount Pleasant, USA; 2 Department of Statistics, Actuarial and Data Science, Central Michigan University College of Medicine, Mount Pleasant, USA

**Keywords:** covid-19, rural america, attitude, media

## Abstract

Background

This study investigated patients’ attitudes about severe acute respiratory syndrome coronavirus 2 in rural Michigan. Despite increasing cases in rural communities across America, surveys have revealed that residents may feel less threatened by the virus compared to their urban counterparts. This difference in attitude and information appraisal can negatively affect rural health by discouraging coronavirus disease (COVID-19) preventative behaviors. Understanding social influences that contribute to the formation of opinions about the pandemic can help public health officials and clinicians better address rural health.

Methodology

This cross-sectional study surveyed 299 participants from three primary care clinics in Shiawassee County of Michigan during a seven-week interval. Statistical analysis, primarily through SAS version 9.4 (SAS Institute Inc., Cary, NC, USA), included descriptive statistics, multiple linear regression models, paired *t-*tests, and correlation coefficients. A *p*-value less than or equal to 0.05 was considered significant.

Results

Patients believed the risk COVID-19 posed to their family was significantly higher than the risk it posed to themselves (*p *< 0.001). Patients who reported that they would follow their provider’s advice for treatment of a non-COVID-19 medical illness were found to be more likely to follow a provider’s advice on COVID-19 (*p *< 0.001). However, patients overall were more agreeable with following provider advice for non-COVID-19 medical illnesses than they were for COVID-19 (*p *< 0.001).

Conclusions

As patients were more agreeable with following medical advice on chronic conditions than COVID-19, there may be extrinsic factors influencing patient views of COVID-19. Polarization of COVID-19 in the media has heavily influenced attitudes toward the virus in America. Initiatives to provide reliable patient education is key to encouraging constructive discussions and a healthy rural community. In a strong patient-provider relationship, primary care providers can share and encourage appropriate healthy behaviors regarding COVID-19, which have a direct impact on community health.

## Introduction

Trends in recent rural studies spurred increasing concern as the weight of the severe acute respiratory syndrome coronavirus 2 (SARS-CoV-2) pandemic shifted from urban to rural settings [1-3]. Rural communities experience disproportionately high rates of comorbidities, including chronic respiratory disease, that increase the chances of contracting coronavirus disease 2019 (COVID-19) [4,5]. As a population known for high rates of comorbidities as well as higher rates of healthcare avoidance compared to the general population, the shifting infectious cases to rural communities became a distinct area of interest [6]. In our study, we assessed patient attitudes concerning SARS-CoV-2, also referred to as COVID-19, in a rural community in Michigan to determine potential contributing factors.

The true reason for the spike in rural communities remains under investigation but is undoubtedly multifaceted. A likely influencing factor includes resident interpretations of the pandemic. In the early stages of the pandemic, there was little difference in perceptions of the virus’s threat based on location. However, between March and June, there was an 11% increase (from 22% to 33%) in rural residents who believed that the threat COVID-19 posed had been blown out of proportion. In addition, there was a 10% drop in the percentage of rural persons who believed the virus to be a major threat to both public health and to themselves in the initial four months [7]. Another study found that, despite reported patient concern, many adults with chronic conditions lacked comprehension about the virus and did not adapt behaviors to prevent the risk of infection [5,8]. In our study, we explored potential factors in Shiawassee County which may have contributed to these trends and possibly a consequent decline in preventative behaviors.

Shiawassee County includes 94.4% white residents with a population density of 133 people/square miles [6]. While 92.4% of the county reports graduating high school, only 17.2% have received a bachelor’s degree or higher [6]. Examining this population’s attitudes may serve as a tool for public health and medical providers to further improve patient education and quality of care across rural populations in general.

## Materials and methods

Participants

This cross-sectional study involving 299 participants was conducted at three Memorial Healthcare primary care sites: Chesaning, Ovid, and Laingsburg. To determine the sample size, we used an established multiple regression result [9]. The coefficients of multiple determination for wearing masks based on demographic variables was 0.45. We assumed that adding the variables of comorbidity and COVID knowledge will increase the coefficients of multiple determination by 0.02. To achieve 80% power with type I error of 0.05, we needed a sample size of 259. A sample size of 299 was determined to ensure the power of the test to be greater than 80%.

Patient participants were enrolled into the study through voluntary response sampling, a nonprobability sampling scheme.

Survey

Patients were offered an in-person, paper questionnaire during office visits. A cover letter was reviewed with each patient and a yes/no question to consent was answered before starting the survey. Verbal consent was accepted as opposed to written consent to avoid collection of personal identifiers. Preventing repetition of patient surveys relied on their recollection of completing the survey in the past. Patients were not offered a survey if they recalled completing a survey at a previous office visit. Participation involved no risk and no incentive was offered. The survey was conducted over seven weeks while investigators were completing medical education at the clinics. The start date of survey dispersal was October 15, 2020 and the end date was December 3, 2020. This anonymous study was determined to be exempt from full board review by the Institutional Review Board at Central Michigan University.

The questionnaire was offered to patients who arrived for a scheduled appointment. The first eight questions covered demographics including sex, age, ethnicity, education level, and history of chronic diseases and COVID-19 infection or contact. The next three inquired what risk level of COVID-19 they perceived in populations. The following seven questions investigated knowledge of COVID-19 Centers for Disease Control and Prevention (CDC) guidelines, as well as their receptiveness toward following their provider’s medical advice. The scale was based on the Severity of Consequences, Standard Linear Scaling by the American Chemical Society [10]. The last question requested the primary source of information regarding COVID-19.

Statistical analyses

Descriptive statistics were provided including mean (standard deviation) for continuous variables and count (percentage) for categorical variables. Multiple linear regression models were used to identify associations between demographic variables and response variables, including risk level of COVID-19 and practices to reduce COVID-19 spreading. The comparisons in means among self, family, and population risk were examined by paired *t-*test, while the associations among self, family, and population risk were examined by correlation coefficients. All of the analytical results were considered to be significant when *p*-values were less than or equal to 0.05. Statistical analysis was primarily conducted using SAS software, version 9.4 (SAS Institute Inc., Cary, NC, USA).

## Results

The final analysis included 299 responses. The study population had an average age 49.2 years with a standard deviation of 18 years. There was a slight male predominance (50.8%), and 94.3% of respondents identified as “white.” The most common level of education marked was “high school graduate” (31.8%). Many patients reported having one or more preexisting conditions, including high blood pressure (34.8%), diabetes (15.4%), being overweight (39.1%), respiratory disease (16.4%), or an immune disorder (8.7%). Overall, 91.0% of the surveyed patients believed they had adequate education on COVID-19. Additionally, 4.0% reported having a positive COVID-19 diagnosis before completing the survey, and 24.7% reported knowing someone in their close contacts who had tested positive.

**Figure 1:**
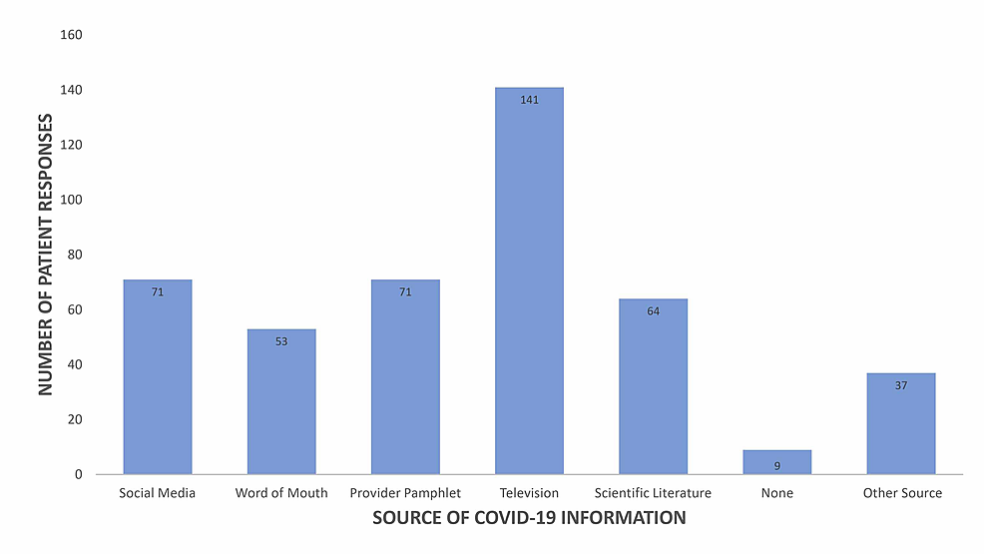
Primary sources of COVID-19 information reported by patients. COVID-19, coronavirus disease 2019

Patients described various primary sources of information about COVID-19 (Figure 1). Patients who did not use social media as a primary source perceived themselves and their families to be at a higher risk from COVID-19 than those who did use social media (Table 1). Patients who reported watching television as their primary source of information perceived the COVID-19 risk level to the population to be higher than those who did not list television as a primary source (Table 1). Additionally, patients who listed watching television as their primary source of information were more likely to agree that social distancing and self-quarantine reduce the spread of COVID-19 (Table 2). Patients who listed scientific literature as their primary source of information were more likely to agree that social distancing, self-quarantine, and hand hygiene prevent the spread of COVID-19 compared with those who did not (Table 2). Patients who used a source to receive COVID-19 information were more likely to have better relationships with their care providers than those who had no source of information (Table 3).

**Table 1:** Relationship between information sources and risk perception with coefficient and estimated p-value using multiple regression model. ref=no, reference equals no in the binary yes/no response

Source	Self-risk		Family risk		Population risk	
	Beta	P-Value	Beta	P-Value	Beta	P-Value
Social media (ref=no)	-0.353	0.023	-0.313	0.037	-0.26	0.06
Word of mouth (ref=no)	0.001	0.994	0.252	0.148	0.088	0.582
Provider pamphlet (ref=no)	-0.099	0.559	-0.118	0.47	-0.041	0.782
Television (ref=no)	0.226	0.122	0.245	0.082	0.274	0.035
Scientific literature (ref=no)	0.125	0.445	0.094	0.552	0.061	0.672
None (ref=no)	0.091	0.812	-0.187	0.612	-0.228	0.502
Other (ref=no)	0.14	0.5	0.071	0.724	-0.031	0.865

**Table 2:** Relationship between information sources and safety behaviors with estimated coefficient and p-value using multiple regression model. ref=no, reference equals no in the binary yes/no response

Source	Masks		Social distance		Self-quarantine		Hand hygiene		
	Beta	P-Value	Beta	P-Value	Beta	P-Value	Beta	P-Value	
Social media (ref=no)	-0.28	0.058	-0.179	0.153	-0.138	0.266	-0.085	0.3	
									Word of mouth (ref=no)	-0.029	0.864	0.036	0.803	0.153	0.29	0.025	0.8		
Provider pamphlet (ref=no)	0.006	0.968	0.066	0.623	0.155	0.247	-0.004	1											
									Television (ref=no)	0.263	0.058	0.284	0.016	0.24	0.04	0.045	0.6		
Scientific literature (ref=no)	0.223	0.148	0.357	0.007	0.261	0.045	0.223	0											
									None (ref=no)	-0.448	0.217	-0.479	0.121	-0.416	0.174	0.083	0.7		
Other (ref=no)	-0.053	0.786	0.19	0.257	0.052	0.756	0.038	0.8											

**Table 3:** Relationship between information sources and trust in provider relationship with estimated coefficient and p-value using multiple regression model. ref=no, reference equals no in the binary yes/no response

Source	Provider relationship trust	
	Beta	P-Value
Social media (ref=no)	0.182	0.05
Word of mouth (ref=no)	-0.116	0.28
Provider pamphlet (ref=no)	0.128	0.198
Television (ref=no)	-0.035	0.689
Scientific literature (ref=no)	0.01	0.92
None (ref=no)	-0.473	0.038
Other (ref=no)	0.075	0.545

**Figure 2:**
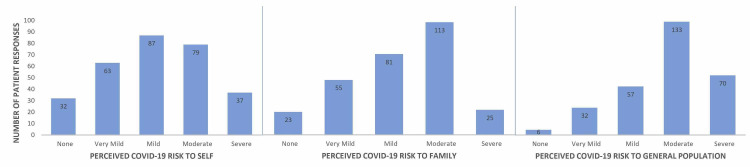
Patients’ perceived COVID-19 risk to themselves, their family, and the general population. COVID-19, coronavirus disease 2019

Patients rated their perceived risk level of COVID-19 to themselves, their family, and the general population (Figure 2). Patients believed the risk that COVID-19 posed to their family was significantly higher than the risk it posed to themselves (*p *< 0.001). They also perceived the risk COVID-19 posed to the general population to be higher than both themselves and their family. Patients who perceived themselves to be at high risk from COVID-19 were more likely to believe the risk level to their family and the general population to be high (*p* < 0.001).

**Figure 3:**
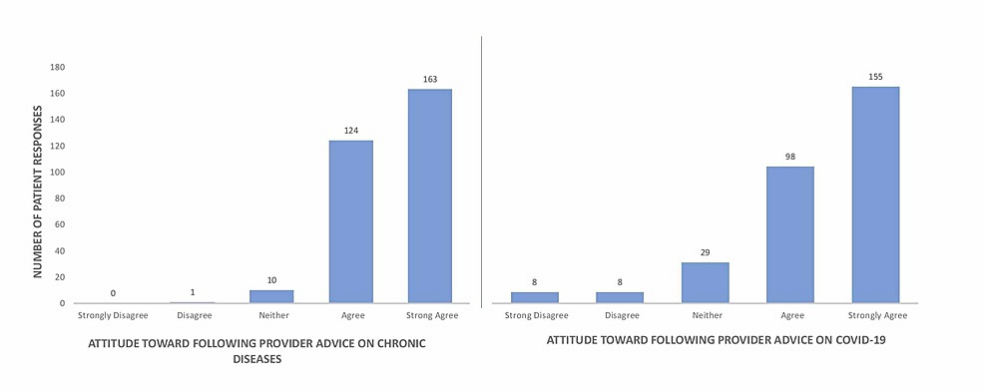
Patients’ attitude toward following provider advice on chronic diseases versus COVID-19. COVID-19, coronavirus disease 2019

Patients also reported their agreeability with following provider advice on both chronic medical conditions and COVID-19 (Figure 3). Patients who reported that they would follow their provider’s advice for treatment of a non-COVID-19 medical illness were found to be more likely to follow a provider’s advice on COVID-19 (*p* < 0.001). However, patients overall were more agreeable with following provider advice for non-COVID-19 medical illnesses than they were for COVID-19 (*p *< 0.001).

## Discussion

Literature suggests that more than half of rural residents are at an increased risk of hospitalization and death if infected with COVID‐19, which puts them at particular risk during this pandemic [11]. Few studies focus on patient’s understanding of COVID-19 in rural America, especially in rural Michigan. By evaluating patient perceptions, we offer insight for improving outcomes at the population level by reducing the risk of transmission through preventative behaviors.

Patients were likely to perceive their family at significantly higher risk of COVID-19 than themselves (Figure 3). Furthermore, they perceived COVID-19 to pose a higher risk to the general population than both themselves and their family, indicating an optimism bias. Optimism bias occurs when individuals believe they are less likely to experience a negative event and more likely to experience a positive event [12]. A study of Americans found overall thoughts about COVID-19 to exhibit unrealistic optimism [13]. For example, people are more likely to believe they won’t become infected. If they do, they believe they are more likely to have a positive outcome. These beliefs may lead to lax adherence to CDC guidelines secondary to lack of perceived consequences [13]. This bias undermines individual motivation to take precautions against COVID-19 [7]. Because optimism bias is an established finding in COVID-19 studies, reducing its influence so that patients appropriately perceive their risk is valuable in a clinical setting. Education about risks to the population using credible sources is the best tool as it can help reduce misconceptions regarding COVID-19 [13]. Informed individuals are more likely to engage in preventive behaviors.

Patients’ attitudes toward COVID-19 are influenced not only by their personal assessment of risk but also by their self-reported knowledge of the virus. Respondents believed they had adequate education about the virus, citing television as the most common source of information (Figure 1). However, patients were more agreeable with following guidelines on other medical illnesses than COVID-19, suggesting they may not view COVID-19 solely from a medical perspective. Recent research looking into the politicization of COVID-19 in the media found that coverage has heavily influenced attitudes toward the virus in America, resulting in potentially skewed perceptions despite a belief of accurate understanding [14]. Augmenting this are results from recent research suggesting that adhering to COVID-19 guidelines is significantly linked to the specific source of one’s local news. The study found that rural residents are more likely to adhere to social distancing guidelines when their local news is produced in an urban area that is affected by COVID-19 [15]. Furthermore, a national study found that national news outlet viewers tended to align their position with the bias of the network, which would translate into their behaviors regarding COVID-19 spread prevention. The study found that respondents who reported watching Fox News were less likely to engage in preventative behaviors and more likely to engage in risky behaviors for COVID-19 compared with those who reported CNN as their primary source of news [16]. Accordingly, patients reporting television as their primary source of information about COVID-19 in our study were found to perceive a higher risk level from COVID-19 to the general population. Overall, further studies are warranted to analyze the effects of COVID-19 coverage on specific networks, channels, or news stations in rural populations in terms of perceptions and behavior.

Although patients were more agreeable to following advice on other medical illnesses than COVID-19, it is important to note that patients were more likely to be open to advice on COVID-19 if they trusted their provider on other medical illness advice. This demonstrates the power of a strong patient-provider relationship at the primary care level. As mentioned previously, there may be several factors molding the attitudes of residents on the pandemic; however, there is an opportunity for providers to convey helpful advice on COVID-19 precautions in the setting of a strong patient-provider relationship.

Limitations should be considered when reviewing the findings of this study. The sample size was limited with 299 participants. The most recent census shows the total population of Shiawassee County as 68,122 people, indicating our study was only able to survey 0.439% of the county’s total population [6]. The survey was distributed in three primary care offices over a two-month period from October to December 2020 at the height of the pandemic. As the pandemic increasingly affected the area, more patients opted for virtual appointments with fewer patients available to survey in person. Patients choosing to meet virtually may have perceived themselves to be at an increased risk, introducing bias in the surveyed population. During the pandemic there was also strict enforcement of mask usage in the primary care office. Patients opposed to wearing a mask may have been discouraged from coming to the doctor, and therefore, may have been underrepresented in the survey. Using paper surveys may have introduced selection bias. As participation in the study was optional, a volunteer bias may also be present.

## Conclusions

Our study aimed to explore patients’ attitudes regarding COVID-19 in rural Shiawassee County, Michigan. The number of positive cases continue to increase in these non-metropolitan areas, posing public health concerns in rural populations as these communities tend to have higher rates of comorbidities compared to their urban counterparts which puts them at a higher risk of mortality from the novel coronavirus. We found that the underlying bias and educational resources can be important influences on attitudes about COVID-19, which ultimately affects behavior. Qualitative research may further illuminate an individual’s multi-faceted rationale for health behaviors and following provider recommendations. Characterizing this information may give insight on how to approach the future impact of public health crises on rural populations. By gaining a better understanding of factors impeding a safety response, we offer an opportunity to improve communication to directly impact the health and well-being of rural residents.
